# Hepatic Lipid Accumulation Alters Global Histone H3 Lysine 9 and 4 Trimethylation in the Peroxisome Proliferator-Activated Receptor Alpha Network

**DOI:** 10.1371/journal.pone.0044345

**Published:** 2012-09-04

**Authors:** Hee-Jin Jun, Jinyoung Kim, Minh-Hien Hoang, Sung-Joon Lee

**Affiliations:** Department of Biotechnology, Graduate School of Life Sciences and Biotechnology, College of Life Sciences and Biotechnology, Korea University, Seoul, Republic of Korea; Karolinska Insitutet, Sweden

## Abstract

Recent data suggest that the etiology of several metabolic diseases is closely associated with transcriptome alteration by aberrant histone methylation. We performed DNA microarray and ChIP-on-chip analyses to examine transcriptome profiling and trimethylation alterations to identify the genomic signature of nonalcoholic fatty liver disease (NAFLD), the most common form of chronic liver disease. Transcriptome analysis showed that steatotic livers in high-fat diet-fed apolipoprotein E2 mice significantly altered the expression of approximately 70% of total genes compared with normal diet-fed control livers, suggesting that hepatic lipid accumulation induces dramatic alterations in gene expression *in vivo*. Also, pathway analysis suggested that genes encoding chromatin-remodeling enzymes, such as jumonji C-domain-containing histone demethylases that regulate histone H3K9 and H3K4 trimethylation (H3K9me3, H3K4me3), were significantly altered in steatotic livers. Thus, we further investigated the global H3K9me3 and H3K4me3 status in lipid-accumulated mouse primary hepatocytes by ChIP-on-chip analysis. Results showed that hepatic lipid accumulation induced aberrant H3K9me3 and H3K4me3 status in peroxisome proliferator-activated receptor alpha and hepatic lipid catabolism network genes, reducing their mRNA expression compared with non-treated control hepatocytes. This study provides the first evidence that epigenetic regulation by H3K9me3 and H3K4me3 in hepatocytes may be involved in hepatic steatosis and the pathogenesis of NAFLD. Thus, control of H3K9me3 and H3K4me3 represents a potential novel NAFLD prevention and treatment strategy.

## Introduction

Histone methylation is one of the most important epigenetic mechanisms for transcriptional regulation of gene expression [1], [2]. Lysine residues in histone proteins, such as H3K4, -9, -27, -36, -79, and H4K20, are favorable sites for mono-, di-, and trimethylation (me1, me2, and me3, respectively) [3], [4]. The degree of methylation at a specific Lys residue in a histone octamer influences the recruitment of effector proteins, thereby affecting chromatin structure and regulating the transcription of downstream genes [5]. Histone methylation regulates a variety of nuclear processes essential for cellular regulation, homeostasis, and fate [5]. Thus, an aberrant histone methylation profile on a genome-wide scale has been associated with many human diseases, such as cancer, through controlling the transcription of downstream target genes [6]–[8].

Nonalcoholic fatty liver disease (NAFLD) occurs in 10–24% of the general population in various countries [9]. Several lifestyle diseases, including metabolic syndrome, obesity, type 2 diabetes, arterial hypertension, and hyperlipidemia, have been shown to be associated with NAFLD [10], [11]. However, the mechanisms involved in the development of NAFLD have not yet been fully clarified. Therefore, a better understanding of the biochemical and pathological changes during the development of NAFLD is needed. The prevalence of hyperlipidemia in patients with NAFLD is 20–92% [9]. Assy et al. reported that approximately half of patients with hyperlipidemia exhibited NAFLD upon ultrasound examination, and hypertriglyceridemia rather than hypercholesterolemia was associated with the risk of NAFLD [12]. Thus, in general, net retention of lipids within hepatocytes, mostly in the form of triglycerides, is a prerequisite for the development of NAFLD. Based on this evidence, NAFLD is characterized by the accumulation of triglyceride within hepatocytes. However, the primary metabolic abnormalities leading to lipid accumulation are not well understood.

Recently, some studies have shown that epigenetic mechanisms are linked to metabolic disorders such as obesity, type II diabetes, hyperlipidemia, atherosclerosis, and cardiovascular disease [13], [14]. Jumonji C-domain-containing histone demethylase 2A (JHDM2a) is an H3K9 demethylase that catalyzes the removal of H3K9 methylation. JHDM2a-knockout mice developed obesity, hypertriglyceridemia, hypercholesterolemia, hyperinsulinemia, and hyperleptinemia with increased body fat deposition and elevated serum lipid levels, regardless of age or food intake [13]. Microarray analysis revealed that JHDM2a deficiency reduced β-oxidation and glycerol release in skeletal muscle [13]. Although histone modification has been suggested to be associated with the development of hyperlipidemia, the association between histone methylation and liver steatosis is largely unknown, and most previous studies were limited to the effect of histone methylation on selected target genes.

In the present study, we investigated variations in H3K9 and H3K4 methylation during the development of NAFLD due to hyperlipidemic conditions by ChIP-on-chip and oligonucleotide DNA array analyses. Hepatic transcriptome profiling with human apolipoprotein E2 (*hAPOE2*) transgenic mice showed that the high-fat diet-induced lipid accumulation responsible for the development of NAFLD altered global gene expression and genes encoding chromatin-remodeling enzymes for H3K9me3 and H3K4me3. Based on the transcriptional regulation, we loaded mouse primary hepatocytes with palmitate plus oleate to induce intracellular lipid-droplet formation to mimic hyperlipidemic NAFLD status, and examined H3K9me3 and H3K4me3 variations in steatotic hepatocytes compared with non-lipid-loaded control cells. Compared with histone mono- and dimethylation, the role of trimethylation in metabolic gene transcription remains poorly understood, even though it has been implicated in the development of several chronic diseases [15], [16]. This study provides the first evidence that the hyperlipidemic condition in hepatocytes induces both hypo- and hypermethylation of H3K9 and H3K4 during NAFLD development.

## Methods

### Animals

Six-week-old male C57BL/6J (Samtako Korea, Osan, Korea) and *hAPOE2* (R158C) transgenic mice (Taconic Farms, Germantown, NY, USA) were used for ChIP-on-Chip and oligonucleotide microarray analyses, respectively. The *hAPOE2* mice were generated by targeted replacement of the endogenous mouse *Apoe* with the human *APOE2* gene and are defective in clearing TG-rich lipoproteins; thus, they spontaneously develop hyperlipidemia [17] and prone to develop hepatic steatosis [18]. Therefore, this mouse strain was appropriate to study gene expression profile for hepatic lipid accumulation. Control diet-fed C57BL/6J and *hAPOE2* mice were maintained on regular rodent chow (12% fat calories, Purina Laboratory Rodent Diet 38057; Dyets Inc., Bethlehem, PA, USA) and high-fat diet-fed *hAPOE2* mice were provided with pelleted rodent chow in which 60% of the calories were from fat (Purina Laboratory Rodent Diet D12492; Dyets Inc.). The animals were maintained with water *ad libitum* on a 12-h light:dark cycle. To obtain liver tissues, mice were killed under general anesthesia with 2.5% tribromoethanol (20 ml/kg, i.p.) and the livers were removed, snap-frozen in liquid nitrogen, and stored at –80°C prior to analysis. All experimental procedures involving mice were approved by the Institutional Animal Care and Use Committee of Korea University (animal protocol number: KUIACUC-20090421-2).

### Preparation of Mouse Primary Hepatocytes and Lipid-loading

Primary hepatocytes of C57BL/6J mice were prepared according a method reported previously [19], [20]. Fasted mice were anesthetized with 2.5% tribromoethanol (20 ml/kg, i.p.), and a catheter was inserted into the inferior vena cava. The superior vena cava was clamped, and the portal vein was transected. The liver was washed with Hanks buffer salt solution (HBSS) containing 100 U/ml penicillin/streptomycin (pH 7.4) for 4 min at a flow rate of 7 ml/min and perfused with HBSS supplemented with 1 mM CaCl_2_ and MgCl_2_, 100 U/ml penicillin/streptomycin, and 0.04% collagenase type IV (pH 7.4) for 10 min. The digested liver was removed and then mechanically disrupted in collagenase solution. The cell suspension was filtered through 70-µm Falcon cell strainers (Falcon BD, Lincoln Park, NJ, USA) and centrifuged at 50× *g* for 2 min. The isolated hepatocytes were washed with phosphate-buffered saline (PBS) by centrifugation at 50× *g* for 2 min. Cells were then cultured on collagen-coated culture plates (Iwaki, Chiba, Japan) in Williams’s Medium E with 10% heat-inactivated fetal bovine serum (FBS), 100 U/ml penicillin/streptomycin, and 1×10^–7^ M insulin for 12 h. Williams’s Medium E was then replaced with low glucose Dulbecco’s modified Eagle’s medium (DMEM) supplemented with 10% FBS and 100 U/ml penicillin/streptomycin. Mouse primary hepatocytes were cultured on collagen-coated culture plates with DMEM containing 10% FBS, 1% penicillin/streptomycin, and 40 µM oleate plus 40 µM palmitate conjugated to 0.16% fatty acid-free bovine serum albumin for 24 h.

### Lipid and Hematoxylin and Eosin (H&E) Staining

For lipid-droplet staining, hepatocytes cultured on collagen-coated glass slides were fixed with 3% (w/v) paraformaldehyde for 30 min and incubated with C_1_-BODIPY 500/510-C_12_ (4,4-difluoro-5-methyl-4-bora-3a,4a-diaza-s-indacene-3-dodecanoic acid; Molecular Probes, Eugene, OR, USA) for 10 min at room temperature. After washing with PBS, coverslips were mounted on slides using the ProLong antifade solution (Invitrogen, Carlsbad, CA, USA) and lipid-droplets in hepatocytes were visualized by fluorescence microscopy (Axio observer D1; Carl Zeiss, Jena, Germany). For H&E staining, the livers of *hAPOE2* mice were fixed with 10% (v/v) formaldehyde, embedded in paraffin, sectioned, and stained with H&E. The tissue sections were observed under microscopy (Eclipse Ti; Nikon Inc, Tokyo, Japan).

### Oligonucleotide Microarray Analysis

Two-color oligonucleotide microarray experiments (n = 6) were performed with the livers of control and high-fat diet-fed *hAPOE2* transgenic mice. Total RNA was extracted from liver tissue using TRIzol reagent (Invitrogen) and further purified using the RNase-free DNase I set and the RNeasy MinElute Cleanup Kit (Qiagen, Chatsworth, CA, USA). cDNA was synthesized from 8 µg purified RNA using Superscript II reverse transcriptase (Invitrogen), oligo(dT)20VN primers, and dNTPs, and subsequently labeled with the Cy3-dUTP and Cy5-dUTP (GE Healthcare, Piscataway, NJ, USA). Labeled cDNA samples were purified using the QIAquick PCR Purification kit (Qiagen) and then hybridized to 38.8 K Mouse Exonic Evidence-Based Oligonucleotide (MEEBO) arrays (Stanford Functional Genomics Facility, Stanford, CA, USA), which contain 30,125 constitutive exonic probes. Hybridized arrays were scanned with the GenePix 4000B scanner (Axon Instruments, Union City, CA, USA) and the resulting images visualized using GenePix 4.0 software (Axon Instruments). Probe-level gene expression values were computed using GenePix 4.0 software and the Stanford Microarray Database. The data were normalized by NormExp background correction (offset = 350) and the Loess and Aquantile methods using the Bioconductor Limma software. Transcriptional responses to high-fat diet feeding were assessed by *p*<0.05 using a *t*-distribution. To analyze the transcriptome profile of steatotic livers, we used the HeatMapViewer included in the GenePattern software (http://genepattern.broadinstitute.org/gp/pages/index.jsf) with gene symbols and log_2_ values of genes regulated significantly. Effects of high-fat diet feeding on biological pathways were assessed by determining the genes significantly regulated using the Database for Annotation, Visualization and Integrated Discovery.

### ChIP-on-chip Analysis

Two-color ChIP-on-chip experiments (n = 2 for each histone status, H3K9me3 and H3K4me3) were performed with non-treated and lipid-accumulated mouse primary hepatocytes isolated from C57BL/6J mice. ChIP was performed using the EZ ChIP kit (Millipore, Billerica, MA, USA) according to the manufacturer’s instructions, with a modification. In brief, mouse primary hepatocytes were fixed with 2% formaldehyde, scraped, collected, and resuspended in SDS lysis buffer supplemented with protease inhibitors. The cells were then sonicated to shear genomic DNA to an average fragment length of 200–1,000 bp, and pelleted by centrifugation at 12,000× *g* for 10 min at 4°C. Chromatins in the supernatant were pre-cleared with protein A-agarose/Salmon Sperm DNA solution. The supernatants underwent overnight immunoprecipitation with chip-grade H3K9me3 (Millipore) and H3K4me3-specific antisera (Millipore). The antibody-associated DNA fragments were recovered using protein A-agarose/Salmon Sperm DNA and eluted with elution buffer (1% SDS and 0.1 M NaHCO_3_). Eluted samples were incubated at 65°C for 4 h to reverse formaldehyde cross-linking and digested with proteinase K (Mbiotech, Seoul, Korea) for 1 h at 45°C to remove proteins. The DNA was extracted using an AxyPrep™ PCR Cleanup Kit (Axygen, Union City, CA, USA). The quality of the ChIPed DNA was determined with a UV-Vis spectrophotometer (NanoDrop Technologies, Wilmington, DE, USA). To amplify genomic DNA, ligation-mediated PCR was performed and PCR product quality was verified by agarose gel electrophoresis (Figure S1). The amplified ChIPed DNA was labeled using a CGH labeling kit (Invitrogen) and hybridized to an Agilent mouse CpG array (105K; Agilent, Santa Clara, CA, USA) using an Oligo aCGH/ChIP-on-Chip Hybridization kit (Agilent). After hybridization, the arrays were washed with an Oligo aCGH/ChIP-on-Chip Wash Buffer kit (Agilent) and scanned using an Agilent DNA microarray scanner (Agilent). Probe-level trimethylation values were computed using Feature Extraction software (Agilent). The data were normalized using the Lowess method with GeneSpring software (Agilent) to remove systematic bias. To identify the targets of differentially trimethylated H3K9 and H3K4 under lipid-accumulated conditions, we set a value of *p*<0.05 as indicating statistical significance assessed using a *t*-distribution and selected targets whose expression was greater than 1.5-fold of the control in at least one histone trimethylation status. Chromosomal distribution of trimethylated targets was visualized with CGH explorer (http://www.ifi.uio.no/forskning/grupper/bioinf/Papers/CGH/). Probe name (Agilent ID) and corresponding chromosome, start position, GenBank accession number, and mean log_2_ values were imported into the software. Biological pathways affected by H3K9me3 and H3K4me3 alterations were clarified by Ingenuity Pathway Analysis (Ingenuity Systems, Redwood City, CA, USA). Agilent ID of the selected targets and their log_2_ trimethylated values were uploaded as the input data set into the software. Biological pathways of H3K9me3 and H3K4me3 were algorithmically generated based on their connectivity, assigned a score, and ranked based on relevance to the input data set in the Ingenuity Pathway Knowledge database. A *p* value for biological pathways was calculated using a right-tailed Fisher’s test. Those biological pathways over a fixed threshold (*p*<0.05) were designated significantly differentially regulated. Based on Gene Ontology annotation, we selected significantly trimethylated candidates associated with lipid metabolism and analyzed their biological relationship, a gene–gene network, using the PubGene database (http://www.pubgene.org/).

### Quantitative Real-time PCR and RT-PCR

Total RNA was extracted from mouse primary hepatocytes using TRIzol reagent (Invitrogen) according to the manufacturer’s instructions. For cDNA synthesis, 2 µg total RNA was reverse-transcribed using oligo(dT) primers, M-MLV reverse transcriptase (Mbiotech), and dNTPs. Levels of gene expression were measured using the iQ5 Real Time PCR Detection System (Bio-Rad, Hercules, CA, USA) and RealMasterMix SYBR ROX (5 PRIME GmbH, Hamburg, Germany). The following thermal conditions were used: 95°C for 3 min, followed by 50 cycles at 95°C for 10 s, 56°C for 15 s, and 72°C for 20 s. Melting curve analysis (71 cycles, starting at 55°C and increasing by 0.5°C every 10 s) was performed to confirm primer specificity. Primers used were designed with OligoPerfect Designer software (Invitrogen). Relative gene expression levels were calculated using the iQ5 Optical System Software version 2 (Bio-Rad), with the expression of each target gene being normalized to that of β-actin. In RT-PCR analysis, synthesized cDNA was used as the template for a standard PCR reaction with PCR-EZ D-PCR Master Mix (Bionics, St. Louis, MO USA). Primer sequences are shown in Table S1.

### ChIP Assay

ChIPed DNA was prepared as described for ChIP-on-chip analysis and used as the template for quantitative PCR reactions. The signal from each sample was normalized to that of the input control. Table S1 lists the sequences of the primers used.

### Statistical Analysis

Data are presented as means ± SEM. Values for two groups were compared using a *t*-test. Differences with *p*<0.05 were considered statistically significant.

## Results

### Expression of Genes Encoding Epigenetic Modifiers is Significantly Altered in the Steatotic Livers of High-fat Diet-fed Human APOE2 Transgenic Mice

**Figure 1:**
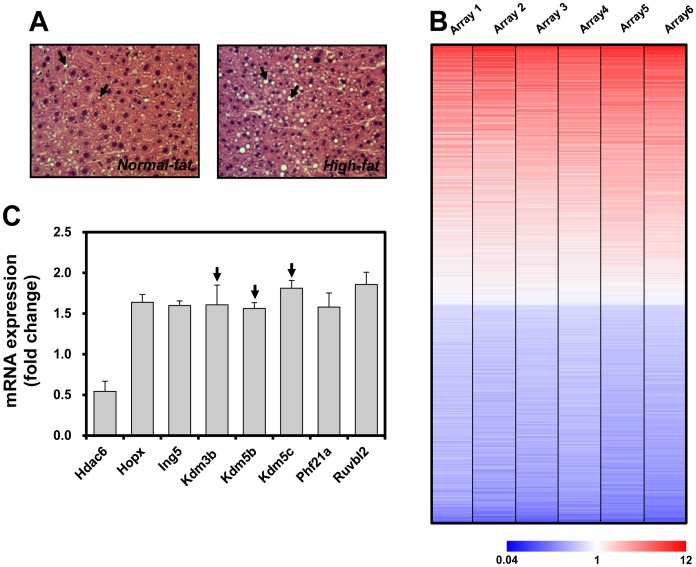
Transcriptome profile of the steatotic livers of high-fat diet-fed *hAPOE2* mice determined by oligonucleotide microarray analysis. (A) H&E staining of the livers of normal diet- and high-fat diet-fed *hAPOE2* mice (original magnification, ×400). (B) Heat map of the transcriptome profile of the steatotic livers of high-fat diet-fed *hAPOE2* mice. Columns represent individual arrays and rows indicate gene expression profiles. Red, blue, and white indicate upregulated, downregulated, and unaltered genes, respectively (*p*<0.05, n = 6). (C) mRNA expression of genes encoding epigenetic modifiers in the steatotic livers of high-fat diet-fed *hAPOE2* mice (*p*<0.05).

We confirmed that high-fat diet feeding exacerbated hepatic lipid accumulation of *hAPOE2* mice compared with that of normal diet-fed *hAPOE2* mice, as assessed by H&E staining (Figure 1A). In hepatic transcriptome analysis, a high-fat diet altered the levels of approximately 70% of the global transcriptome of the livers of *hAPOE2* mice compared with those of normal diet-fed *hAPOE2* mice (Figure 1B). Pathway analysis of genes whose expression was greater than 1.5-fold of control (*p*<0.05) suggested that high-fat diet feeding induced alterations in chromatin modification-related genes. In particular, we found that genes encoding enzymes for regulation of H3K4me3 and H3K9me3, such as the jumonji C-domain-containing histone demethylase (JHDM) family (*Kdm3b*, *Kdm5b*, *Kdm5c*), were significantly induced in high-fat fed livers compared with control livers (Figure 1C). Accordingly, we further investigated whether hepatic lipid accumulation could lead to aberrant H3K4me3 and H3K9me3, and eventually contribute to development of NAFLD. To test this hypothesis, we examined global H3K4me3 and H3K9me3 alterations in lipid-accumulated mouse primary hepatocytes by ChIP-on-chip analysis.

### Lipid Accumulation Induces Genome-wide H3K9me3 and H3K4me3 Variations in Mouse Primary Hepatocytes

**Figure 2:**
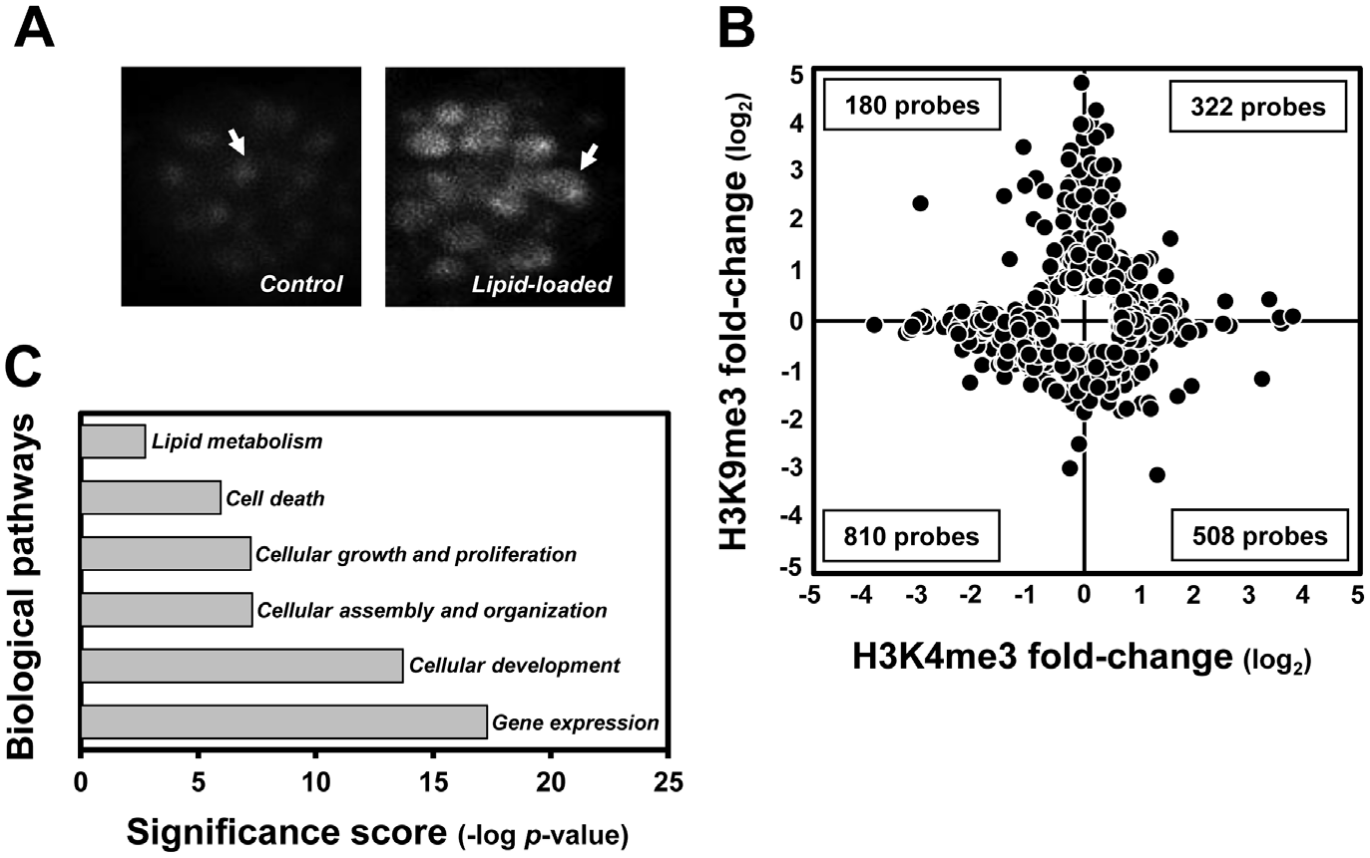
Genome-wide H3K9me3 and H3K4me3 variations in lipid-accumulated mouse primary hepatocytes determined by ChIP-on-chip analysis. (A) BODIPY-labeled lipid droplets in non- and palmitate plus oleate-treated mouse primary hepatocytes. (B) Expression pattern of H3K9me3 and H3K4me3 targets (fold change ≥1.5 in at least one histone trimethylation status, *p*<0.05, n = 2 for each histone status, H3K9me3 and H3K4me3) in lipid-accumulated hepatocytes. (C) Biological pathways affected by H3K9me3 and H3K4me3 targets in response to lipid accumulation in hepatocytes (*p*<0.05).

We first isolated primary hepatocytes from C57BL/6J mice livers (Figure S2A) and verified the expression of albumin and transferrin, hepatocyte-specific markers (Figure S2B) [21]. To induce lipid accumulation in primary hepatocytes for mimicking steatotic liver, palmitate and oleate were treated and then lipid droplet formation in the hepatocytes was verified (Figure 2A). The expressions of the jumonji C-domain-containing histone demethylase (JHDM) family (*Kdm3b*, *Kdm5b*, *Kdm5c*) were significantly induced in lipid accumulated primary hepatocytes compared with those in control cells (Table S2) We performed ChIP-on-chip analysis to investigate the H3K9me3 and H3K4me3 alterations caused by lipid accumulation in mouse primary hepatocytes. A total of 1,830 targets on the 405 K CpG array displayed a ≥1.5-fold change of at least one histone trimethylation status, showing that target hyper- and hypotrimethylated H3K9me3 and H3K4me3 were evenly chromosomally distributed in lipid-loaded hepatocytes, with diverse signal intensities (Figure S3); 332 and 810 targets were commonly up- or downregulated in both H3K9me3 and H3K4me3, respectively. However, 688 targets showed an inverse H3K9 and H3K4 trimethylation pattern: a total of 180 targets displayed increased H3K4me3 and decreased H3K9me3, whereas 508 exhibited the opposite pattern (Figure 2B). These target genes had many biological functions, including gene expression, cellular development, assembly/organization, growth/proliferation, cell death, and lipid metabolism, as assessed by Ingenuity Pathway Analysis (Figure 2C). Furthermore, hepatic lipid accumulation induced H3K9me3 and H3K4me3-related alterations in the expression of genes associated with liver hepatomegaly, proliferation, steatohepatitis, necrosis, hyperplasia, hyperproliferation, steatosis, regeneration, dysplasia, hypertrophy, damage, and degeneration, all of which are associated with the pathophysiology of NAFLD, although the number of targeted genes was small and thus the pathway was not significant (data not shown).

### Lipid Accumulation Specifically Alters the Effect of H3K9me3 and H3K4me3 on the Peroxisome Proliferator-activated Receptor Alpha (PPARα)-network

**Figure 3:**
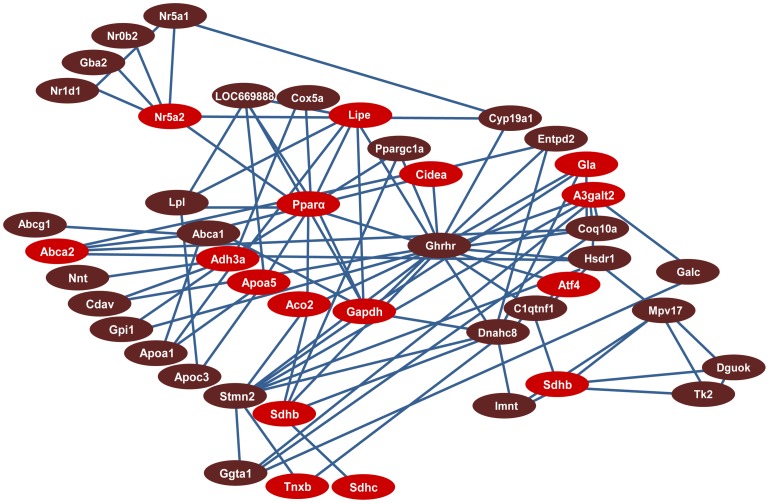
Effect of H3K4me3 and H3K9me3 on the PPARα-network in lipid-accumulated mouse primary hepatocytes. Lipid metabolism-associated H3K9me3 and H3K4me3 targets (*p*<0.05) were selected based on Gene Ontology annotation, and their biological relationship was analyzed using the PubGene database. Red indicates H3K9me3 and H3K4me3 targets detected by ChIP-on-chip analysis and dark red represents those genes possessing a potential biological relationship with targets detected by ChIP-on-chip analysis based on previous reports.

Based on the ChIP-on-chip data, we selected 22 lipid metabolism genes with altered H3K9me3 and H3K4me3 status in the promoter, as identified by Gene Ontology annotation (Table S3). We then investigated whether these genes were involved in cellular metabolic pathways associated with NAFLD development. A PubGene analysis for biological networks revealed that 16 of the 22 gene targets were biologically closely associated. In particular, we found that *Pparα* and related lipid catabolism genes, including *Apoa5*, nuclear receptor subfamily 5, group A, member 2 (*Nr5a2*), lipase, hormone-sensitive (*Lipe*), isocitrate dehydrogenase 3 (NAD^+^) alpha (*Idh3α*), aconitase 2 (*Aco2*), activating transcription factor 4 (*Atf4*), succinate dehydrogenase complex, subunit B, iron sulfur (*Sdhb*), cell death-inducing DNA fragmentation factor, and alpha subunit-like effector A (*Cidea*), were associated with an altered H3K9me3 or H3K4me3 status in lipid-accumulated hepatocytes (Figure 3 and Table S3).

**Figure 4:**
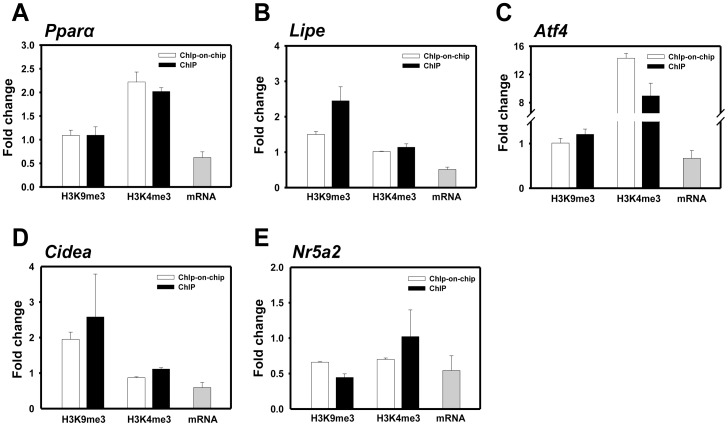
ChIP analysis of mRNA levels of H3K9me3 and H3K4me3 and selected *Pparα* network genes (n = 3).

We selected five genes, *Pparα*, *Nr5a2*, *Lipe*, *Atf4*, and *Cidea*, which are involved in hepatic lipid metabolism, and validated the ChIP-on-chip results with a conventional ChIP assay. The histone status of the selected genes determined by a conventional ChIP assay was similar to the results of the ChIP-on-chip analysis (Figure 4). In addition, since histone methylation plays a key role in the transcriptional regulation of gene expression, we also determined mRNA expression of *Pparα* and its related genes selected in the ChIP-on-chip analysis. Quantitative real-time analysis showed reduced expression of all five genes in lipid-accumulated hepatocytes compared with non-treated control cells (Figure 4). This may induce defects in lipid catabolism under hyperlipidemic conditions and eventually contribute to the development of NAFLD.

## Discussion

NAFLD refers to accumulation of hepatic steatosis not due to excess alcohol consumption [9] and is the most common liver condition globally [22]. The pathogenesis of NAFLD is related to increased lipid influx into the liver and increased *de novo* hepatic lipogenesis, promoting hepatic triglyceride accumulation [9]. Defects in lipid utilization via mitochondrial oxidation and lipid export may also contribute to hepatic lipid buildup [9]. Subjects with NAFLD have a higher mortality rate than the general population and are at increased risk of developing cardiovascular disease and diabetes [23]. Histologically, NAFLD occurs as a spectrum from mild hepatic steatosis only, to nonalcoholic steatohepatitis characterized by hepatocellular injury and inflammation, to cirrhosis [9]. The molecular epigenetic mechanisms behind each symptom have begun to be investigated. In this study, we aimed to identify molecular markers altered in lipid-loaded hepatocytes in the early stage of NAFLD.

Metabolic pathway analysis using transcriptome data revealed that genes encoding chromatin-remodeling enzymes, such as jumonji C-domain-containing histone demethylases that regulate H3K9me3 and H3K4me3 [24], were significantly altered in steatotic livers. Thus, we further investigated the genome-wide H3K9me3 and H3K4me3 status in primary hepatocytes from C57BL/6J mice by ChIP-on-chip analysis.

For ChIP-on-chip analysis, isolated mouse primary hepatocytes were incubated with palmitate plus oleate to induce accumulation of lipid droplets and mimic hyperlipidemic conditions. This is a widely used method of inducing cellular lipid droplet formation [25]. H3K9me3 and H3K4me3 variations in lipid-loaded cells were compared to non-lipid-loaded control hepatocytes.

Lipid accumulation in the ChIP-on-chip assay mimics a relatively early stage of NAFLD, which ranges from simple hepatic steatosis to a potentially progressive form, nonalcoholic steatohepatitis, and the highest end of the severity spectrum, cirrhosis [9]. Thus, our findings suggest that H3K9 and H3K4 methylation status of genes involved in steatosis and steatohepatitis may be associated with early-stage NAFLD. Additionally, *Pparα*, phosphatase and tensin homolog (*Pten*), cyclin-dependent kinase inhibitor 1A (*Cdkn1a*), spectrin beta, non-erythrocytic 1 (*Sptbn1*), and mediator complex subunit 1 (*Med1*), all of which are involved in biological pathways responsible for the development of NAFLD, exhibited altered H3K9me3 and H3K4me3 status in lipid-loaded hepatocytes.

Histone modification plays a key role in gene transcription by inducing changes in chromatin structure. In general, euchromatin states lead to gene expression, while heterochromatin states facilitate gene silencing. Early studies showed that H3K9me3 is largely associated with heterochromatin and gene silencing [26], while H3K4me3 is linked to euchromatin [27], where actively transcribed genes are located, with exceptions such as Vakoc, the transcription of which is associated with H3K9 trimethylation [28]. The biological functions of histone methylation with regard to gene promoters and coding regions remain incompletely understood. Thus more research, especially on a genomic scale, is required to better understand histone trimethylation, such as H3K4me3 and H3K9me3. According to early studies of histone methylation and target gene expression, the H3K9me3 and H3K4me3 profile should overlap only slightly; however, over 1,000 genes were affected by both H3K9 and H3K4 hypo- or hypertrimethylation in our ChIP-on-chip results. In addition, many genes affected by hypertrimethylated H3K4 were not influenced by H3K9me3. Moreover, this pattern was evident in lipid metabolism- and hepatic steatohepatitis-related genes selected according to Gene Ontology (*p*<0.05).

Note that under hyperlipidemic conditions, the influence of H3 trimethylation on *Pparα* was mirrored by other lipid catabolism-related genes, including *Lipe*, *Atf4*, *Nr5a2*, and *Cidea*, all of which are associated with the pathophysiology of NAFLD. Moreover, the expression of all of these genes was moderately reduced in lipid-loaded primary hepatocytes with marked H3K4 or H3K9 hypertrimethylation. First, *Pparα* is highly expressed in the liver and is a critical transcription factor responsible for the regulation of hepatic lipid accumulation, modulating target genes related to fatty acid oxidation, including carnitine palmitoyltransferase 1 (*Cpt1*), acetyl-CoA synthase and acyl-CoA oxidase, fatty acid synthesis, and triglyceride hydrolysis [29]. Thus, *Ppara*-null mice were reportedly defective in fatty acid utilization and displayed a fatty liver phenotype [30], and activation of PPARα with an agonist compound has been suggested as a therapeutic strategy for NAFLD [31]. Second, *Lipe* promotes hepatic lipid oxidation *in vivo*. Overexpression of hepatic *Lipe* induces fatty acid oxidation and eventually ameliorates steatosis [32]. Third, *Atf4* also influences fatty acid oxidation by regulating *Cpt1* and medium chain acyl-CoA dehydrogenase in the liver [33]. Fourth, *Nr5a2* is an activator of cholesterol 7 alpha-hydroxylase for cholesterol excretion [34]. Finally, the metabolic function of *Cidea* in hepatocytes is not clearly understood, although its expression was reduced in the livers of type 2 diabetic mice exhibiting steatosis [35]. All these suggest that aberrant modulation of PPARα and its related genes associated with lipid catabolism at an epigenetic level may contribute to hepatic lipid accumulation and eventually the development of NAFLD. The underlying cause of liver fat accumulation in NAFLD is mostly due to the inhibition of fatty acid catabolism, in which PPARα has been continuously suggested as a target molecule for pathogenesis and treatment of NAFLD [36]. Indeed, in also human study, mRNA expression levels of *Pparα* was suppressed in both the livers of obese and non-obese patients with NAFLD compared with normal controls [37]. Therefore, to study and establish whether the reduction of *Pparα* mRNA in NAFLD patients could be induced by aberrant modulation at an epigenetic level, as we showed in this study, the results found in the current study are needed to be replicate, further investigated, and expanded in the livers of NAFLD patients.

We performed experiments with primary hepatocytes in conventional monolayer to investigate the sole effects on the hepatocytes, which is a major cell type responsible for hepatic lipid metabolism. Results suggested that the isolated primary hepatocytes express high levels of transferrin and albumin, the hepatocyte-specific genes, assessed with RT-PCR (Figure S2B) indicating the cell preparation was done appropriately. Previously reported data suggested that gene expression levels in primary hepatocytes are somewhat different from those in the liver. However, when compared with cultured hepatoma cell lines, primary hepatocytes overall show much similarity in gene expression profile to those of the livers [38]. Several factors may affect the gene expressions in primary cells. First, the genes primarily expressed in nonparenchymal cells, such as genes in inflammatory reaction genes including iNOS, TNFα, IL-1β, IL-10, are not highly expressed in primary hepatocytes [39]. Second, the three-dimensional architecture and the extracellular matrix are not well maintained in primary cells thus mRNA expression of collagen and other structural proteins is generally decreased [20]. Additionally, the expression of P450 is known to diminish in vitro over culture time [40]. However, it is widely accepted that the lipid metabolism and related gene expressions in primary hepatocytes are quite similar to the livers thus primary cells may be appropriate system for the purpose, although not perfect [20], [38].

The current study provides the first evidence of genome-wide histone trimethylation changes in response to hyperlipidemic conditions during the development of NAFLD. This has potential applications in the development and assessment of drugs and nutrients for NAFLD therapeutics.

## Supporting Information

Figure S1 **ChIP verification.** After H3K9me3- and H3K4me3-specific immunoprecipitation, chromatin fragmentation and random-prime amplification of genomic DNA fragments were performed, resulting in production of high-quality DNA for labeling reactions and microarray hybridization.
(TIF)

Figure S2 **Primary hepatocytes isolated from C57BL/6J mice.** Primary hepatocytes were obtained by perfusion of the livers of C57BL/6J mice with collagenase type IV. (A) Photomicrograph of a monolayer of the isolated primary mouse hepatocytes (magnification, ×40). (B) mRNA levels of the typical hepatocyte markers albumin and transferrin assessed by RT-PCR analysis.
(TIF)

Figure S3 **Chromosomal distribution of H3K9me3 and H3K4me3 targets.** Each dot indicates the mean log2 value of signals in the H3K9- or H3K4-trimethylated region of their corresponding gene.
(TIF)

Table S1 **Primers used in the conventional ChIP assay, quantitative real-time PCR, and RT-PCR.**
(DOC)

Table S2 **Relative expression of genes encoding epigenetic modifiers in the primary hepatocyte loaded with palmitate and oleate.**
(DOC)

Table S3 **Twenty-two H3K9me3 and H3K4me3 lipid metabolism targets.**
(DOC)
