# Correction: Hepatic Lipid Accumulation Alters Global Histone H3 Lysine 9 and 4 Trimethylation in the Peroxisome Proliferator-Activated Receptor Alpha Network

**DOI:** 10.1371/annotation/eff6e471-306a-41bd-88e3-13857af094af

**Published:** 2012-11-30

**Authors:** Hee-Jin Jun, Jinyoung Kim, Minh-Hien Hoang, Sung-Joon Lee

The following information was missing from the Funding section: This work was conducted with support from the Cooperative Research Program for Agriculture Science & Technology Development (Project No. 201203013026150010400 and No 201203010306090010400), Rural Development Administration, Suwon, Republic of Korea and the Basic Science Re- search Program of the National Research Foundation of Korea (NRF), funded by the Ministry of Education, Science and Technology (20100028180). 

