# Supplementary figures and images for: Hepatic Lipid Accumulation Alters Global Histone H3 Lysine 9 and 4 Trimethylation in the Peroxisome Proliferator-Activated Receptor Alpha Network
Source: PLoS One. 2012 Sep 4;7(9):e44345. doi: 10.1371/journal.pone.0044345 (PMC3433434; doi:10.1371/journal.pone.0044345)

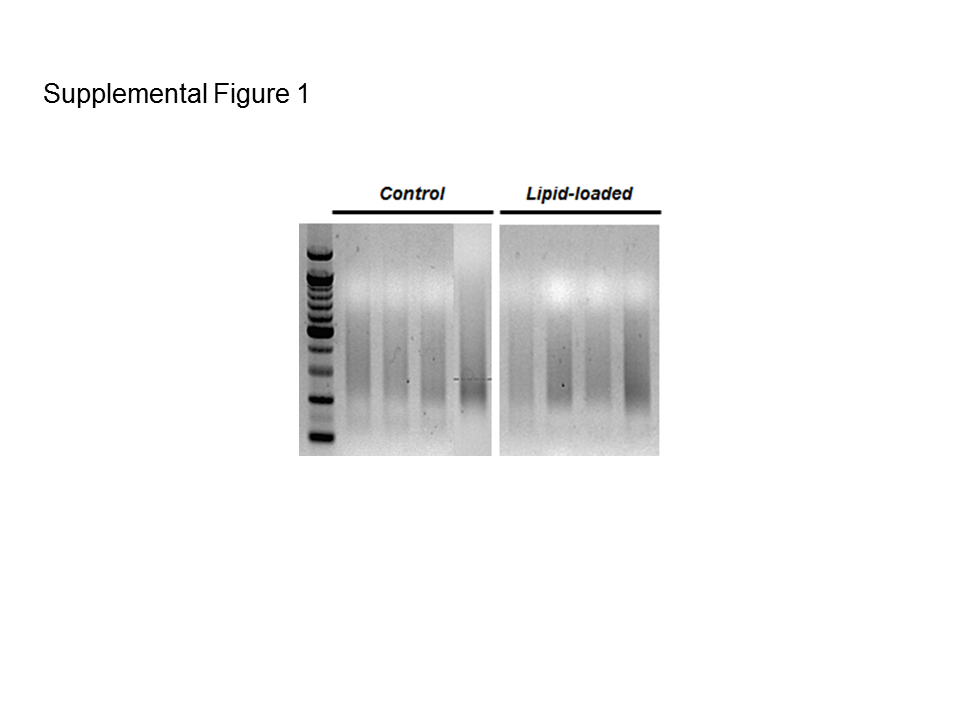

Supplement: Figure S1 — ChIP verification. After H3K9me3- and H3K4me3-specific immunoprecipitation, chromatin fragmentation and random-prime amplification of genomic DNA fragments were performed, resulting in production of high-quality DNA for labeling reactions and microarray hybridization. (TIF) [file pone.0044345.s001.tif]

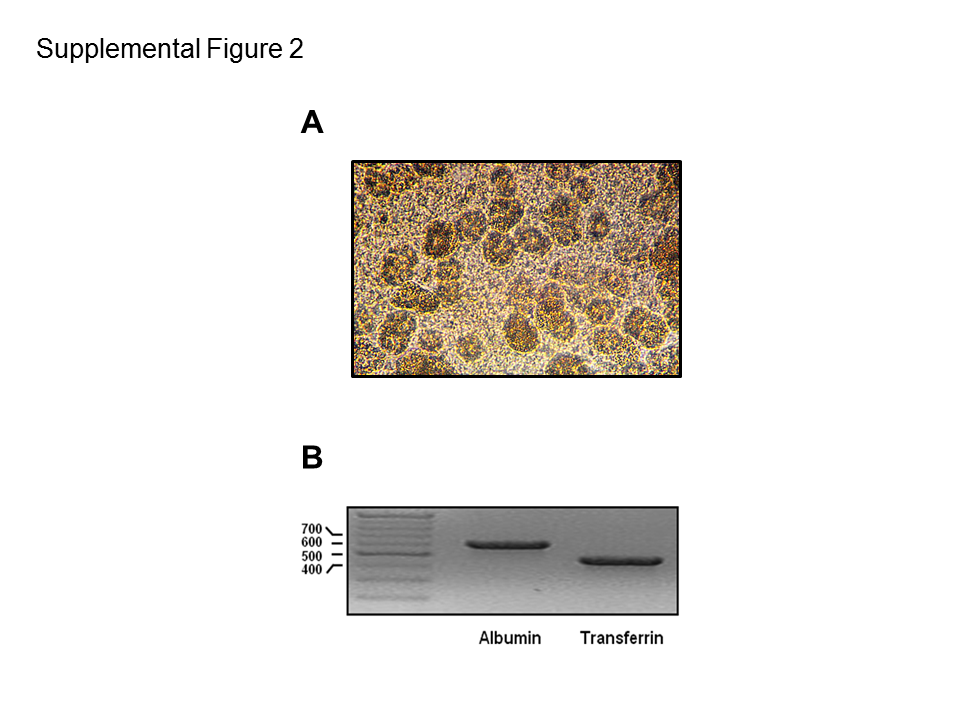

Supplement: Figure S2 — Primary hepatocytes isolated from C57BL/6J mice. Primary hepatocytes were obtained by perfusion of the livers of C57BL/6J mice with collagenase type IV. (A) Photomicrograph of a monolayer of the isolated primary mouse hepatocytes (magnification, ×40). (B) mRNA levels of the typical hepatocyte markers albumin and transferrin assessed by RT-PCR analysis. (TIF) [file pone.0044345.s002.tif]

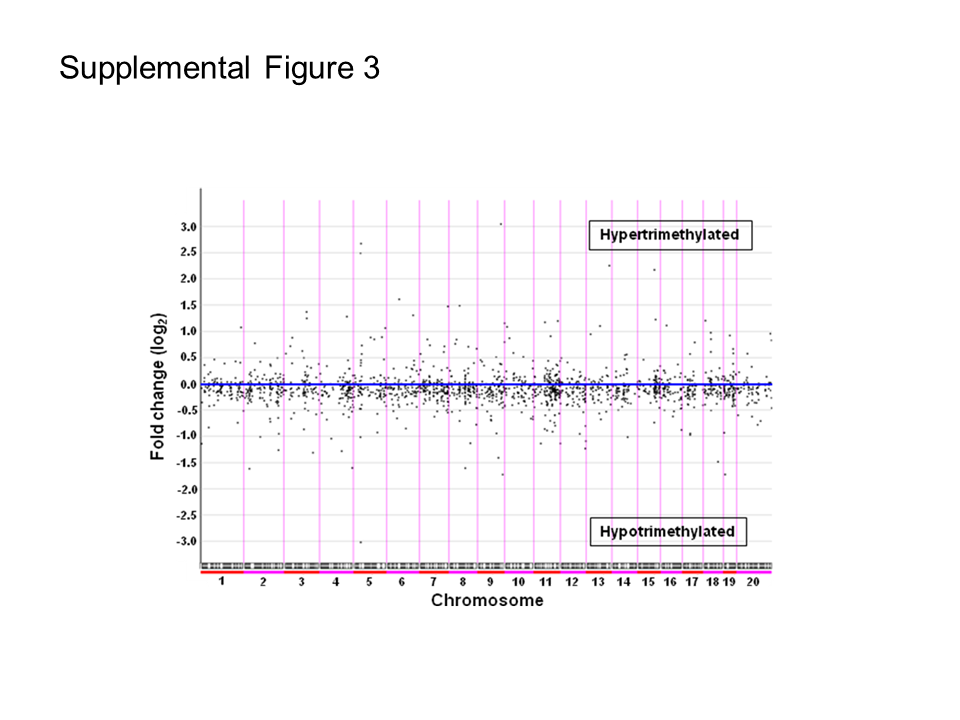

Supplement: Figure S3 — Chromosomal distribution of H3K9me3 and H3K4me3 targets. Each dot indicates the mean log2 value of signals in the H3K9- or H3K4-trimethylated region of their corresponding gene. (TIF) [file pone.0044345.s003.tif]
