# Supplementary material for: Hepatic Lipid Accumulation Alters Global Histone H3 Lysine 9 and 4 Trimethylation in the Peroxisome Proliferator-Activated Receptor Alpha Network
Source: PLoS One. 2012 Sep 4;7(9):e44345. doi: 10.1371/journal.pone.0044345 (PMC3433434; doi:10.1371/journal.pone.0044345)
Supplement: Table S1 — Primers used in the conventional ChIP assay, quantitative real-time PCR, and RT-PCR. (DOC) [file pone.0044345.s004.doc]

**Table S1.** Primers used in the conventional ChIP assay, quantitative real-time PCR, and RT-PCR

| **Gene** | **Primer**  **(5’ to 3’)** | **Sequence** |
| --- | --- | --- |
| **Primers used in conventional ChIP assay** | | |
| *Pparα* | Sense  Antisense | AGGGATGAGTGTGGCTAACCT  TCAGAGGGTACATGCCTGACT |
| *Lipe* | Sense  Antisense | CCCTCCCTCCTCTTAGGAGAT  CATCTCTCCAGCTCTGTTTGG |
| *Atf4* | Sense  Antisense | GGGAACACAGTGACCCTTGTA  ATTCTACCAGCAGGCAAGTGA |
| *Cidea* | Sense  Antisense | AGCCTTACTCGGGAAGACAAG  TAAGGGGAACATTCGTTGACA |
| *Nr5a2* | Sense  Antisense | GGTGTTCCAAGCCACTGATAA  CATTGGGTGATTCTTTCTCCA |
| **Primers used in quantitative real-time PCR** | | |
| β-actin | Sense  Antisense | TGCTGTCCCTGTATGCCTCT  AGGTCTTTACGGATGTCAACG |
| *Pparα* | Sense  Antisense | AAGAACCTGAGGAAGCCGTTCTGT  GCAGCCACAAACAGGGAAATGTCA |
| *Lipe* | Sense  Antisense | ATTCGCCATAGACCCAGAGTT  CCTTCCCGTAGGTCATAGGAG |
| *Atf4* | Sense  Antisense | CTCACTGGCGAGTGTAAGGAG  ACAAGCACAAAGCACCTGACT |
| *Cidea* | Sense  Antisense | AGTCTGCAAGCAACCAAAGAA  GATGGCTGCTCTTCTGTATCG |
| *Nr5a2* | Sense  Antisense | TACCATTACGGTCTCCTCACG  GAATGAGGGCTTTCTTCTGCT |
| **Primers used in RT-PCR** | | |
| Albumin | Sense  Antisense | GCACACAAGAGTGAGATVGC  TCTGCATACTGGAGCACTTC |
| Transferrin | Sense  Antisense | GTATTATGCCGTGGCTGTGG  GCTTGGGCCAGGTGGCAG |
