# Supplementary material for: Hepatic Lipid Accumulation Alters Global Histone H3 Lysine 9 and 4 Trimethylation in the Peroxisome Proliferator-Activated Receptor Alpha Network
Source: PLoS One. 2012 Sep 4;7(9):e44345. doi: 10.1371/journal.pone.0044345 (PMC3433434; doi:10.1371/journal.pone.0044345)
Supplement: Table S2 — Relative expression of genes encoding epigenetic modifiers in the primary hepatocyte loaded with palmitate and oleate. (DOC) [file pone.0044345.s005.doc]

**Table S2.** Relative expression of genes encoding epigenetic modifiers in the primary hepatocyte loaded with palmitate and oleate.

| **Gene** | Relative Gene Expression* |
| --- | --- |
| *Hdac6* | 0.75±0.17 |
| *Hopx* | 1.25±0.28 |
| *Ing5* | 1.73±0.17 |
| *Kdm3b* | 2.56±0.16 |
| *Kdm5b* | 2.78±0.11 |
| *Kdm5c* | 2.45±0.20 |
| *Phf21a* | 3.02±0.12 |
| *Ruvbl2* | 1.89±0.11 |

*, relative gene expressions in lipid-loaded primary hepatocytes compared with those in the control primary cells.
