# Supplementary material for: Hepatic Lipid Accumulation Alters Global Histone H3 Lysine 9 and 4 Trimethylation in the Peroxisome Proliferator-Activated Receptor Alpha Network
Source: PLoS One. 2012 Sep 4;7(9):e44345. doi: 10.1371/journal.pone.0044345 (PMC3433434; doi:10.1371/journal.pone.0044345)
Supplement: Table S3 — Twenty-two H3K9me3 and H3K4me3 lipid metabolism targets. (DOC) [file pone.0044345.s006.doc]

**Table S3.** Twenty-two H3K9me3 and H3K4me3 lipid metabolism targets

| **Chromosome** | **Start** | **End** | **Accession number** | **Gene name** | **Gene symbol** | **Fold change** | |
| --- | --- | --- | --- | --- | --- | --- | --- |
|  |  |  |  |  |  | **K9** | **K4** |
| 15 | 81699941 | 81699985 | NM_080633 | aconitase 2, mitochondrial | Aco2 | 2.23 | 2.34 |
| 5 | 30444686 | 30444730 | NM_178878 | hydroxyacyl-coenzyme A dehydrogenase/3-ketoacyl-coenzyme A thiolase/enoyl-coenzyme A hydratase (trifunctional protein), alpha subunit | Hadha | 2.09 | 0.95 |
| 14 | 72287125 | 72287169 | NM_011506 | succinate-coenzyme A ligase, ADP-forming, beta subunit | Sucla2 | 2.02 | 0.49 |
| 2 | 25250890 | 25250934 | NM_007379 | ATP-binding cassette, subfamily A (ABC1), member 2 | Abca2 | 2.00 | 0.33 |
| 7 | 79988880 | 79988924 | NM_173011 | isocitrate dehydrogenase 2 (NADP^+^), mitochondrial | Idh2 | 1.96 | 0.70 |
| 18 | 67469132 | 67469176 | NM_007702 | cell death-inducing DNA fragmentation factor, alpha subunit-like effector A | Cidea | 1.95 | 0.87 |
| 2 | 5868587 | 5868631 | NM_001081131 | dehydrogenase E1 and transketolase domain-containing 1 | Dhtkd1 | 1.58 | 0.83 |
| 9 | 54384446 | 54384490 | NM_029573 | isocitrate dehydrogenase 3 (NAD^+^) alpha | Idh3a | 1.52 | 0.84 |
| 7 | 25104334 | 25104378 | NM_001039507 | lipase, hormone-sensitive | Lipe | 1.50 | 1.02 |
| X | 129947568 | 129947617 | NM_013463 | galactosidase, alpha | Gla | 0.95 | 14.51 |
| 1 | 172986936 | 172986981 | NM_025321 | succinate dehydrogenase complex, subunit C, integral membrane protein | Sdhc | 1.02 | 10.12 |
| 4 | 151022454 | 151022498 | NM_133348 | acyl-CoA thioesterase 7 | Acot7 | 0.99 | 3.88 |
| 15 | 85563696 | 85563740 | NM_011144 | peroxisome proliferator-activated receptor alpha | Pparα | 1.09 | 2.22 |
| 6 | 118527631 | 118527676 | NM_001081112 | ankyrin repeat domain 26 | Ankrd26 | 0.54 | 1.01 |
| 6 | 125130469 | 125130513 | NM_008084 | Glyceraldehyde 3-phosphate dehydrogenase | Gapdh | 0.89 | 2.08 |
| 3 | 82989749 | 82989793 | NM_023624 | lecithin-retinol acyltransferase (phosphatidylcholine-retinol-*O*-acyltransferase) | Lrat | 0.55 | 0.68 |
| 4 | 128269882 | 128269927 | NM_001009819 | alpha 1,3-galactosyltransferase 2 (isoglobotriaosylceramide synthase) | A3galt2 | 0.57 | 0.63 |
| 9 | 46021040 | 46021084 | NM_080434 | apolipoprotein A-V | Apoa5 | 0.60 | 0.90 |
| 4 | 140233770 | 140233814 | NM_023374 | succinate dehydrogenase complex, subunit B, iron sulfur (Ip) | Sdhb | 0.64 | 0.78 |
| 1 | 138765281 | 138765325 | NM_030676 | nuclear receptor subfamily 5, group A, member 2 | Nr5a2 | 0.66 | 0.70 |
| 17 | 34288271 | 34288315 | NM_031176 | tenascin XB | Tnxb | 0.68 | 0.62 |
| 15 | 80083399 | 80083448 | NM_009716 | activating transcription factor 4 | Atf4 | 1.01 | 14.29 |
